# mzMatch–ISO: an R tool for the annotation and relative quantification of isotope-labelled mass spectrometry data

**DOI:** 10.1093/bioinformatics/bts674

**Published:** 2012-11-17

**Authors:** Achuthanunni Chokkathukalam, Andris Jankevics, Darren J. Creek, Fiona Achcar, Michael P. Barrett, Rainer Breitling

**Affiliations:** ^1^College of Medical Veterinary and Life Sciences, Institute of Molecular Cell and Systems Biology, University of Glasgow, Glasgow G12 8QQ, UK, ^2^Groningen Bioinformatics Center, Groningen Biomolecular Sciences and Biotechnology Institute, University of Groningen, 9747 AG Groningen, The Netherlands, ^3^Department of Biochemistry and Molecular Biology, Bio21 Molecular Science and Biotechnology Institute, University of Melbourne, Victoria 3010, Australia, ^4^Wellcome Trust Centre for Molecular Parasitology, College of Medical Veterinary and Life Sciences, Institute of Infection, Immunity and Inflammation, University of Glasgow, Glasgow G12 8TA, UK and ^5^Faculty of Life Sciences, Manchester Institute of Biotechnology, University of Manchester, Manchester M1 7DN, UK

## Abstract

**Motivation:** Stable isotope**-**labelling experiments have recently gained increasing popularity in metabolomics studies, providing unique insights into the dynamics of metabolic fluxes, beyond the steady-state information gathered by routine mass spectrometry. However, most liquid chromatography–mass spectrometry data analysis software lacks features that enable automated annotation and relative quantification of labelled metabolite peaks. Here**,** we describe mzMatch–ISO, a new extension to the metabolomics analysis pipeline mzMatch.R.

**Results:** Targeted and untargeted isotope profiling using mzMatch–ISO provides a convenient visual summary of the quality and quantity of labelling for every metabolite through four types of diagnostic plots that show (i) the chromatograms of the isotope peaks of each compound in each sample group; (ii) the ratio of mono-isotopic and labelled peaks indicating the fraction of labelling; (iii) the average peak area of mono**-**isotopic and labelled peaks in each sample group; and (iv) the trend in the relative amount of labelling in a predetermined isotopomer. To aid further statistical analyses, the values used for generating these plots are also provided as a tab**-**delimited file. We demonstrate the power and versatility of mzMatch–ISO by analysing a ^13^C**-**labelled metabolome dataset from trypanosomal parasites.

**Availability:** mzMatch.R and mzMatch–ISO are available free of charge from http://mzmatch.sourceforge.net and can be used on Linux and Windows platforms running the latest version of R.

**Contact:** rainer.breitling@manchester.ac.uk **.**

**Supplementary information:** Supplementary data are available at *Bioinformatics* online

## 1 INTRODUCTION

Liquid chromatography–mass spectrometry (LC–MS) is a technique that combines the physical separation capabilities of liquid chromatography with the highly sensitive mass detection properties of mass spectrometry. Metabolomics studies use LC–MS for the global detection and relative quantification of metabolites in complex biological samples. Recently, LC–MS has been applied to trace the metabolism of stable isotope-labelled metabolic precursors in biological systems as a function of time (Supplementary Fig. S1) (Chaneton *et al.*, 2012; Le *et al.*, 2012). Such experiments can provide unique insights into the dynamics of metabolic fluxes, beyond the steady-state information gathered by routine metabolomics.

**Fig. 1.**
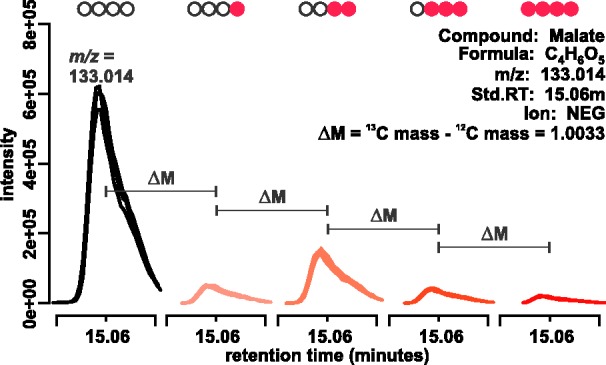
A representative example of an unlabelled peak (first peak) and its corresponding ^13^C-labelled peaks. These peaks elute at the same retention time, but their masses differ by the difference in the mass of heavy and light carbon. Filled circles show the number of labelled carbons that each peak represent

Stable isotope-labelled metabolites possess the same chromatographic properties as their unlabelled counterparts and can be readily identified from the MS dataset based on their expected mass (Fig. 1). However, a key challenge that metabolomics researchers face is the limited number of suitable bioinformatic solutions for metabolome-wide isotope-labelled data analyses. Multiple MS data analysis tools are available (Dunn *et al.*, 2012), including widely used open source software such as mzMine (mzmine.sourceforge.net), mzMatch (mzmatch.sourceforge.net) and XCMS (metlin.scripps.edu), and commercial software such as SIEVE (www.thermo.com), MassHunter (www.chem.agilent.com) and MarkerLynx (www.waters.com). Although they are all capable of identifying and quantifying metabolites of interest in unlabelled data, features that enable the extraction and relative quantification of isotope peaks from labelled data either require manual intervention or are non-existent. Furthermore, software that can handle labelled MS data, such as MetExtract (Bueschl *et al.*, 2012) and MAVEN (Melamud *et al.*, 2010), lacks appropriate peak-picking algorithms in the processing pipeline. Here, we present a novel and unique tool, mzMatch–ISO, that circumvents these bottlenecks by performing fully automated targeted or untargeted annotation and relative quantification of mono-isotopic and corresponding isotope-labelled peaks of metabolites in stable isotope-labelled LC–MS data to generate plots and tables that describe the labelling pattern in detail.

The ability to generate a comprehensive graphical visualization of the output along with the extensible and scriptable nature of the software itself makes mzMatch–ISO a unique data analysis tool for isotope-labelling studies. mzMatch–ISO is an extension to mzMatch, an open-source Java toolbox for MS data processing and visualization (Scheltema *et al.*, 2011). Features of mzMatch—enabled by the R package mzMatch.R—including the new PeakML data exchange format and the data analysis pipeline were described by Scheltema *et al.* (2011). mzMatch has been applied to many metabolomics data analyses (Jankevics *et al.*, 2011; t’Kindt *et al.*, 2010) and is the underlying platform for software such as IDEOM (Creek *et al.*, 2012). Currently, only LC–MS data analysis is supported by mzMatch–ISO; however, it is possible to process gas chromatography–mass spectrometry data analyses using mzmatch.R and mzmatch–ISO with some additional scripting.

## 2 METHODS

Isotope profiling using mzMatch–ISO requires the LC–MS raw data (.mzXML) files to be preprocessed by extracting the peaks using XCMS (Smith *et al.*, 2006) and aligning, noise filtering and gap-filling peaks into a combined PeakML file containing all samples using mzMatch.R. In addition to the PeakML file, targeted profiling requires an additional tab-delimited input text file containing the list of compounds of interest (see Supplementary Fig. S2). This list can also contain characteristic adducts or fragments of metabolites of interest where appropriate. Automated targeted and untargeted isotope profiling can be performed using the mzMatch–ISO functions PeakML.Isotope.TargettedIsotopes() and PeakML.Isotope.UntargettedIsotopes(), respectively. The latter can be used for profiling global label distribution by looking for the isotopes of all identified peaks in a PeakML file; or all compounds in databases such as the Kyoto Encyclopedia of Genes and Genomes (KEGG) (Kanehisa *et al.*, 2012) or the Human Metabolome Database (HMDB) (Wishart *et al.*, 2009); or using common metabolic transformations recursively, as described in Breitling *et al.* (2006), Gipson *et al.* (2008), Rogers *et al.* (2009) and Weber and Viant (2010). All parameters used in these functions are described at http://mzmatch.sourceforge.net/isotopes-targetted.php.

**Fig. 2.**
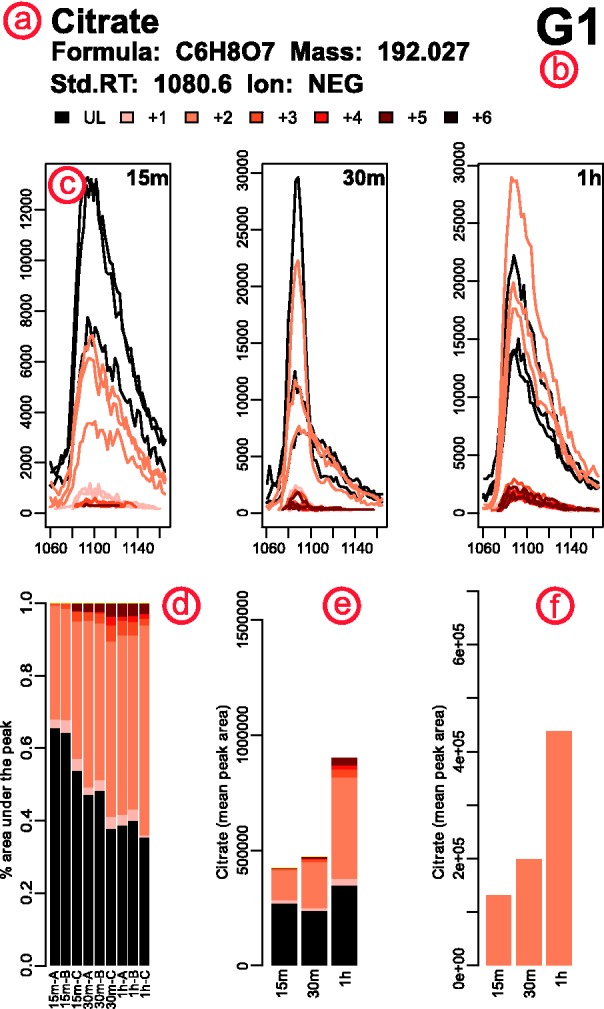
Representative example of the output PDF file generated by mzMatch–ISO (see text for details)

For both targeted and untargeted analysis, mzMatch–ISO generates two outputs—a PDF file and a tab-delimited file. The former contains one page per metabolite with various plots that describe the pattern of labelling observed (Fig. 2). The page header shows compound information from the target list or the database used for identification, and the ionisation polarity (Fig. 2a). In cases where more than one peakset is present within a given mass window, each peakset is plotted on a separate page of the PDF file (Fig. 2b); usually the correct peakset can be identified by considering the retention time and intensity profile.

Chromatograms of each unlabelled peak and its corresponding labelled isotopomers for each sample in each condition are shown (Fig. 2c), the peak area/height being stored in the accompanying tab-delimited file. The intensity and shape of the chromatograms helps to assess the effect of noisy or incomplete peaks on the reported pattern of labelling. Furthermore, these chromatograms can be used along with the plot in Figure 2d, showing the normalized peak area/height of each mono-isotopic peak and its isotopomers in each replicate, to make informed decisions on outliers by assessing the variability in labelling between replicates. The overall trend in the labelling pattern of a metabolite, as observed between various conditions involved in the study, is also visualized (Fig. 2e). This plot is especially useful in time–series analyses to rapidly observe the dynamics of relative concentration changes. The final plot (Fig. 2f) not only highlights the labelling trend of an isotopomer of choice, but, in the case of one-carbon labelling studies, it also compares observed signals with the theoretical intensity expected based on the natural abundance of the relevant isotope. This plot is of extreme importance in studies where natural abundance has to be distinguished from low levels of labelling.

## 3 RESULTS

The automated untargeted isotope annotation and relative quantification capabilities of mzMatch–ISO are demonstrated by the analysis of LC–MS data from procylic form *Trypanosoma brucei* grown on ∼50% uniformly ^13^C-labelled glucose medium for 5 days. The plot in Supplementary Figure S3 generated from the tab-delimited output file (data are provided in Supplementary file S4, and the scripts are available on the website) highlights the capabilities of mzMatch–ISO in demonstrating a complex biological phenomenon.

mzMatch–ISO provides an efficient and user-friendly output for the analysis and compact visualization of isotope-labelled metabolomics datasets without the need for specialist bioinformatics skills, allowing rapid, precise and meaningful biological interpretation. The algorithm can be implemented directly in R, or from the IDEOM graphical user interface, to facilitate follow-up statistical processing, analyses and re-plotting of the results.

*Funding*: A.C. was funded by a Scottish Universities Life Sciences Alliance (SULSA) grant to R.B. Funding for A.J. was provided by a Netherlands Organisation for Scientific Research NWO-Vidi grant to R.B. D.J.C. was supported by an Australian National Health and Medical Research Council postdoctoral training fellowship. F.A. was supported by SysMO, NWO-Vidi and SULSA. M.P.B. was supported by the Wellcome Trust through The Wellcome Trust Centre for Molecular Parasitology, which is supported by core funding from the Wellcome Trust [085349].

*Conflict of Interest*: none declared.

## Supplementary Material

Supplementary Data
