# Supplementary material for: mzMatch–ISO: an R tool for the annotation and relative quantification of isotope-labelled mass spectrometry data
Source: Bioinformatics. 2012 Nov 17;29(2):281–3. doi: 10.1093/bioinformatics/bts674 (PMC3546800; doi:10.1093/bioinformatics/bts674)
Supplement: Supplementary Data [file supp_29_2_281__index.html]

mzMatch–ISO: an R tool for the annotation and relative quantification of isotope-labelled mass spectrometry data — Supplementary Data 

# mzMatch–ISO: an R tool for the annotation and relative quantification of isotope-labelled mass spectrometry data

## Supplementary Data

files

**Files in this Data Supplement:**

- Supplementary Data - pdf file
- Supplementary Data - pdf file
- Supplementary Data - xls file
