# Supplementary material for: mzMatch–ISO: an R tool for the annotation and relative quantification of isotope-labelled mass spectrometry data
Source: Bioinformatics. 2012 Nov 17;29(2):281–3. doi: 10.1093/bioinformatics/bts674 (PMC3546800; doi:10.1093/bioinformatics/bts674)
Supplement: Supplementary Data [file supp_bts674_supplementary.pdf]

Supplementary Figure 1

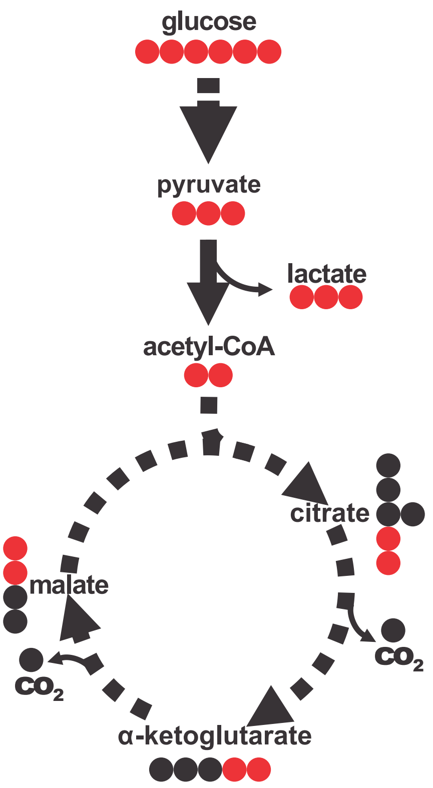

Figure S1: Tracing the distribution of heavy isotope labelling in metabolites constituting a pathway. Red and black filled circles represent labelled ^{13}C carbon and unlabelled ^{12}C carbon, respectively.

Supplementary Figure 2

id	name	formula	rt	follow
GLU	Glucose	C6H12O6	13.56	6
G6P	Glucose-6-phosphate	C6H13O9P		6
GAP	Glyceraldehyde-3-phosphate	C3H7O6P		3
⋮	⋮	⋮		

Figure S2: Tab–delimited input file required for targeted isotope profiling containing the details of the compounds of interest. A unique identifier that distinguishes the compound, the name of the compound and its formulae as shown in the first three columns are mandatory. Providing standard retention time (in the 'rt' column), if available, can aid in the precise identification of mono–isotopic peaks and corresponding isotopic peaks for the compound of interest. To enable this feature an appropriate retention time window has to be specified in the 'stdRTWindow' parameter of the PeakML.Isotope.TargettedIsotope() function. The last column, 'follow', can be used to specify the isotopomer that has to be tracked by generating a separate plot in the PDF output (6: track the isotopomer with 6 heavy atoms, etc.).

Supplementary Figure 3

Figure S3: A flux map generated as a result of an automated untargeted annotation and quantification of isotope profiles of compounds in an LC–MS dataset from procylic form *Trypanosoma brucei* grown on ∼50% uniformly ^{13}C-labelled glucose medium for five days. The good match between expected (P) and observed (M) isotopomer patterns confirm the performance achieved by the automated mzMatch–ISO pipeline. Raw data from LC–MS experiments were initially converted to .mzXML files using the ReAdW tool from the Trans-Proteomic Pipeline (TPP) and then subjected to the automated mzMatch processing pipeline as described in http://mzmatch.sourceforge.net/tutorial.mzmatch.r.advanced.php. The PeakML file after combining based on conditions, filtering and gap–filling was profiled for isotopes of all compounds in the KEGG database using the PeakML.Isotope.UntargettedIsotopes() function using a 5–ppm mass window. Data from the tab–delimited output file was used to generate the heat map for the measured values in the plot. The predicted labelling patterns of the metabolites were calculated based on a binomial distribution considering the expected maximal number of atoms obtained from glucose and from other unlabelled sources for each metabolite. The glucose pool was considered to contain 50% labelled and 50% unlabelled glucose (with natural abundance of ^{13}C) (Murphey, B.F. and Nier, A.O. (1941) Variations in the Relative Abundance of the Carbon Isotopes. *Phys. Rev.*, 59(771)). Ribose 5-phosphate (R5P) is obtained from the pentose phosphate pathway (PPP) and hydrolysis of unlabelled nucleosides present in the medium. The complex exchanges of carbons in the PPP make it difficult to predict the R5P labelling pattern. Therefore, the predicted pattern of R5P was based on the measured pattern of S-Methyl-5-Thio-D-Ribose 1-Phosphate, the labelled carbons of which, can only come from R5P. Subsequently, the labelling patterns of all metabolites derived from R5P were calculated based on this pattern. Metabolites in bold letters are external, i.e., they do not contain atoms derived from labelled glucose, but only the natural abundance of ^{13}C.

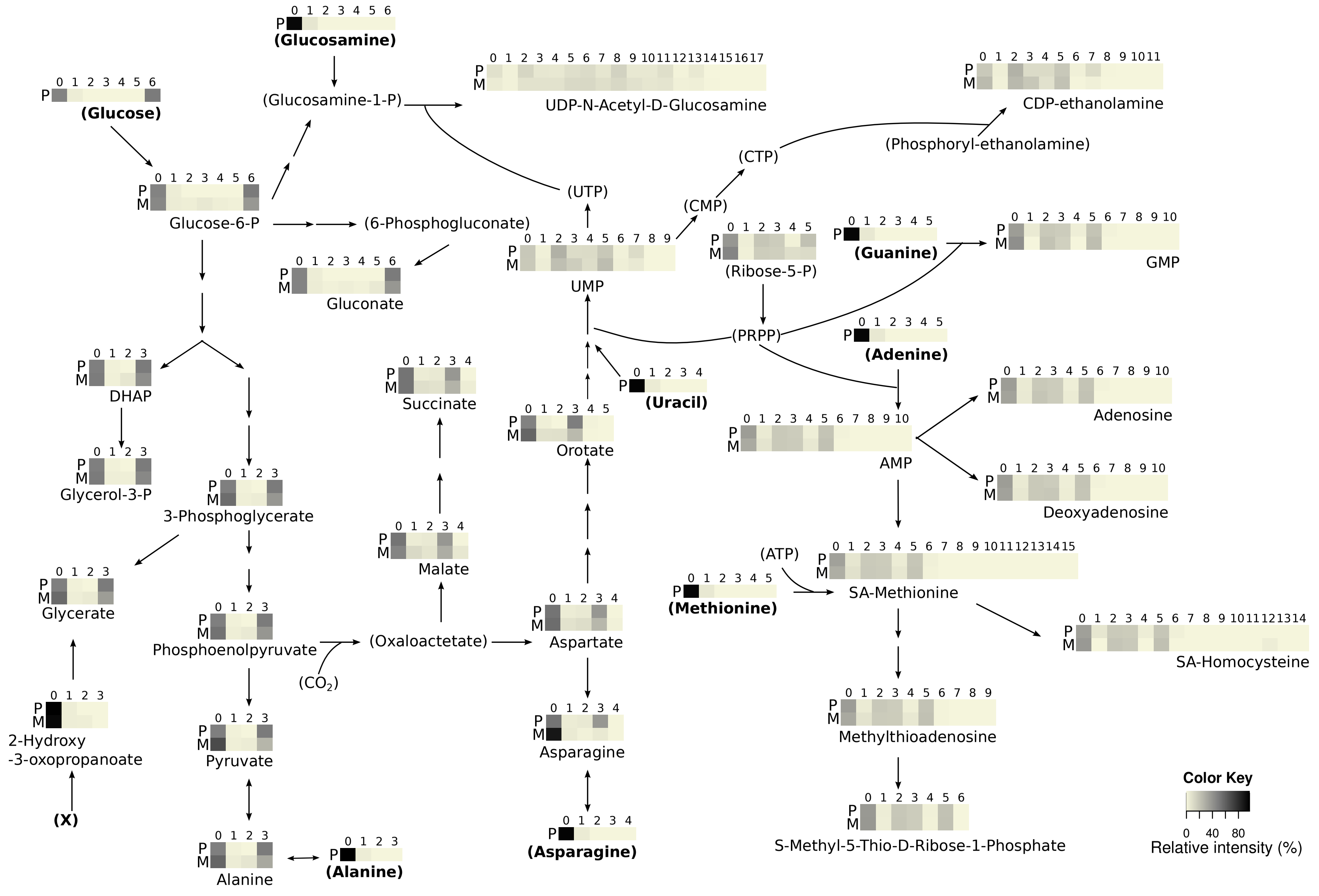
(Glucose)
(Glucosamine)
(Glucosamine-1-P)
UDP-N-Acetyl-D-Glucosamine
CDP-ethanolamine
(Phosphoryl-ethanolamine)
(CTP)
(CMP)
(UTP)
Glucose-6-P
(6-Phosphogluconate)
Gluconate
UMP
(Ribose-5-P)
(PRPP)
(Guanine)
GMP
(Adenine)
Adenosine
AMP
Deoxyadenosine
DHAP
Glycerol-3-P
3-Phosphoglycerate
Succinate
Malate
Orotate
(Uracil)
Glycerate
Phosphoenolpyruvate
(CO$_2$)
(Oxaloactetate)
Aspartate
Asparagine
(Asparagine)
(ATP)
(Methionine)
SA-Methionine
SA-Homocysteine
Methylthioadenosine
S-Methyl-5-Thio-D-Ribose-1-Phosphate
2-Hydroxy
-3-oxopropanoate
(X)
Pyruvate
Alanine
(Alanine)
Color Key
Relative intensity (%)

Figure S3:
